# Supplementary material for: Image-Based Dosimetry in Dogs and Cross-Reactivity with Human Tissues of IGF2R-Targeting Human Antibody
Source: Pharmaceuticals (Basel). 2023 Jul 8;16(7):979. doi: 10.3390/ph16070979 (PMC10384855; doi:10.3390/ph16070979)
Supplement: Supplementary file 1 [file pharmaceuticals-16-00979-s001.zip › pharmaceuticals-2475152-supplementary.pdf]

Table S1. Cross Reactivity of B-IF3with Normal Human Tissues

Tissue	Source	Run	Test Article (B-IF3)		Control Article (B-hIgG1)		Assay Control	Tissue Validation (Tissue Staining) Control	Tissue Comments/Nonspecific Findings
			10 µg/mL	1 µg/mL	10 µg/mL	1 µg/mL			
**Skin**	HT 551-6	1,2						Pos	Endogenous pigment (melanin).
Epithelial cells, epidermis, sebaceous and/or sweat glands (cytoplasmic granules)			1+ (rare)	1+ (very rare)	Neg	Neg	Neg		Mainly located in the basal layer of the epidermis.
Mononuclear leukocytes (cytoplasm/cytoplasmic granules)			1-2+ (rare to occas)	1-2+ (rare)	Neg	Neg	Neg		Mainly located surrounding hair follicles.
Other elements			Neg	Neg	Neg	Neg	Neg		
**Small intestine**	HT 2391-1	1,2						Pos	Residual endogenous peroxidase (rare resident leukocytes).
Epithelial cells, mucosa (cytoplasm/cytoplasmic granules)			1+ (rare)	1+ (rare)	Neg	Neg	Neg		Mainly located in epithelial cells near the surface.
Neurons (perikaryon/perikaryonic granules)			1+ (occas to freq)	1+ (occas)	Neg	Neg	Neg		Located in ganglia.
Other elements			Neg	Neg	Neg	Neg	Neg		

Table S1 (continued). Cross Reactivity of B-IF3with Normal Human Tissues

Tissue	Source	Run	Test Article (B-IF3) 10 µg/mL	Test Article (B-IF3) 1 µg/mL	Control Article (B-hIgG1) 10 µg/mL	Control Article (B-hIgG1) 1 µg/mL	Assay Control	Tissue Validation (Tissue Staining) Control	Tissue Comments/Nonspecific Findings
**Spleen**	HT 1910-18	1,2						Pos	Residual endogenous peroxidase (rare resident leukocytes). Endogenous pigment (hemosiderin). In slide 1, there was smudgy to granular staining of very rare spindle cell focally in the capsule that was of uncertain specificity and section of capsule was not present on slides 2-5.
Mononuclear leukocytes (cytoplasm/cytoplasmic granules)			1+ (rare)	1+ (very rare)	Neg	Neg	Neg		
Reticulo-endothelial cells (cytoplasm/cytoplasmic granules)			1-2+ (freq)	1+ (occas)	Neg	Neg	Neg		
Other elements			Neg	Neg	Neg	Neg	Neg		
**Stomach**	HT 1906-12	1,2						Pos	Residual endogenous peroxidase (rare resident leukocytes).
Epithelial cells, mucosa (cytoplasm/cytoplasmic granules)			1+ (rare)	1+ (very rare)	Neg	Neg	Neg		Mainly located in epithelial cells near the surface.
Neurons (perikaryon/perikaryonic granules)			1+ (rare)	1+ (very rare)	Neg	Neg	Neg		Located in ganglia.
Other elements			Neg	Neg	Neg	Neg	Neg		

± = equivocal, 1+ = weak, 2+ = moderate, 3+ = strong, 4+ = intense, Neg = Negative, Pos = Positive, M = Missing, NE = Not Evaluated, NS = Not Stained, occas = occasional, freq = frequent. Frequency modifiers were included to provide the approximate percentage staining of expected numbers of that cell type or tissue element at that location. The frequency of cells with staining was identified as follows: very rare (<1% of cells of a particular cell type); rare (1-5% of cells of a particular cell type); rare to occasional (>5-25% of cells of a particular cell type); occasional (>25-50% of cells of a particular type); occasional to frequent (>50-75% of cells of a particular cell type); frequent (>75-100% of cells of a particular cell type).
